# Effects of mobilisation with movement (MWM) on anatomical and clinical characteristics of chronic ankle instability: a randomised controlled trial protocol

**DOI:** 10.1186/s12891-019-2447-x

**Published:** 2019-02-13

**Authors:** Ishanka Weerasekara, Peter Grant Osmotherly, Suzanne Jordan Snodgrass, John Tessier, Darren Anthony Rivett

**Affiliations:** School of Health Sciences, Faculty of Health and Medicine, The University of Newcastle, Callaghan, Australia

**Keywords:** Ankle sprain, Chronic ankle instability, Joint mobilisation, Mobilisation with movement, Fibular positional fault, Mechanical instability, Functional instability

## Abstract

**Background:**

Up to 40% of individuals who sprain their ankle develop chronic ankle instability (CAI). One treatment option for this debilitating condition is joint mobilisation. There is preliminary evidence that Mulligan’s Mobilisation With Movement (MWM) is effective for treating patients with CAI, but the mechanisms by which it works are unclear, with Mulligan suggesting a repositioning of the fibula. This randomised controlled trial aims to determine the effects of MWM on anatomical and clinical characteristics of CAI.

**Methods:**

Participants 18 years or over with CAI will be accepted into the study if they satisfy the inclusion and exclusion criteria endorsed by the International Ankle Consortium. They will be randomised into the experimental group (MWM) or the placebo group (detuned laser) and will receive the assigned intervention over 4 weeks. General joint hypermobility and the presence of mechanical instability of the ankle will be recorded during the first visit. Further, position of the fibula, self-reported function, ankle dorsiflexion range, pressure pain threshold, pain intensity, and static and dynamic balance will be assessed at baseline, and at the conclusion of course of intervention. Follow-up data will be collected at the twelfth week and at the twelfth month following intervention.

**Discussion:**

Effectiveness of MWM on clinically relevant outcomes, including long term benefits will be evaluated. The capacity of MWM to reverse any positional fault of the fibula and the association of any positional fault with other clinically important outcomes for CAI will be explored. Proposed biomechanical mechanisms of fibular positional fault and other neurophysiological mechanisms that may explain the treatment effects of MWM will be further explored. The long term effectiveness of MWM in CAI will also be assessed.

**Trial registration:**

Australian New Zealand Clinical Trials Registry; ACTRN12617001467325 (17/10/2017).

## Background

Up to 40% of patients with an initial ankle sprain develop chronic ankle instability (CAI), which is frequently associated with recurrent sprains and persistent pain [1, 2]. A recurrent subjective perception of the ankle joint ‘giving way’ is clinically indicative of CAI [3], which is defined as “repetitive bouts of lateral ankle instability resulting in numerous ankle sprains” [4]. Clinical management of CAI often involves balance and sport-related activity training [5]. In a recent meta-analysis, preliminary evidence was also found supporting joint mobilisation as a clinically effective intervention in improving dynamic balance and dorsiflexion range of motion in CAI [6].

Several ankle joint mobilisation procedures have been developed and described by renowned manual therapists such as Geoffrey Maitland, Freddy Kaltenborn and Brian Mulligan, and are commonly used in rehabilitation [7]. These procedures are applied to a joint, either in the form of non-thrust passive joint mobilisations, high velocity thrust manipulation, or Mobilisation With Movement (MWM). MWM is defined as the application of a sustained passive accessory movement to a joint while the patient actively performs a task/movement that was previously identified as being painful or limited [8]. After manual application of MWM, tape is applied to help maintain the glide and corrected fibular position [9]. Biomechanically and neurophysiologically mediated mechanisms have been proposed to explain how these joint mobilisation procedures may work [8, 10]. The proposed neurophysiological mechanisms are based on animal [11] and human experiments [12] related to pain science and motor systems [8]. These have shown that joint manual therapy techniques including MWM, activate a descending pain inhibitory pathway which is non-opioid mediated [8]. One proposed biomechanical mechanism relates to a reduction of an entrapped meniscoid or synovial fringe by a specifically directed MWM glide particularly in those instances where only one repetition is required to bring about a substantial and long lasting effect [8].

Of our recent systematic review and meta-analysis identified greater effects for MWM and Mulligan taping compared to Maitland joint mobilisation (with and without traction) and joint thrust manipulation [6, 13]. Dorsiflexion range of motion and self-reported instability were some of the outcomes for which there was evidence of improvement from MWM, although the long term benefits were unclear [14–16]. Most of the previous studies on chronic ankle sprains have applied MWM to the talocrural joint [14–18], and few studies have applied MWM taping [19–21]. However Mulligan proposes that an anterior fibular positional fault commonly results from ankle inversion sprains, and that a MWM using a posterior glide of the fibula to correct this should be trialled after 48 h following such an injury [22]. Patients with recurrent ankle sprains may also benefit from this MWM treatment combined with taping aimed at maintaining the posterior fibula glide, with reportedly less ‘giving way’ and greater confidence in using the ankle in patients with functional instability and pain [22]. Therefore this study is designed to evaluate the clinical benefits of the fibular posterior glide MWM with Mulligan taping, and whether it corrects any demonstrable positional fault which may exist. The prevalence of pain in people with CAI is high (60.1%) [23] and to our knowledge no studies have assessed the effect of MWM on pain. In addition, the present study will assess the effects of MWM on pressure pain threshold in CAI. The presence of localized peripheral sensitization has been previously identified in acute inversion ankle sprains [24] and in subacute ankle sprains [25]. Balance impairments in CAI are frequently reported in the literature and MWM has been found to be effective immediately after application, but there is presently insufficient research to determine the short term benefits of MWM for balance impairments [6]. The present study plans to address this deficiency in the literature as well.

A positional fault at the inferior tibio-fibular joint, is one arthrokinematic abnormality proposed to be related to persistent/recurrent symptoms and repetitive ankle sprains in CAI [4]. In the case of an ankle joint sprain, Mulligan suggests that the distal fibula is ‘mal-positioned’ anteriorly (anterior positional fault) following an inversion injury and that chronicity may result if this remains uncorrected [22, 26]. Preliminary evidence for such an anterior fibular positional fault was identified in Hubbard et al’s study of individuals with CAI [27]. However it is unclear whether ankle instability caused the anterior fibular position or whether the fault itself was actually the predisposing factor to re-injury. Also, the clinical importance of an anterior fibular positional fault in relation to other potential contributors to CAI remains unclear. Further, the mechanism(s) of changes in CAI outcomes after MWM needs to be further investigated [15, 28]. It has been proposed by Mulligan in his positional fault hypothesis, that MWM effects an immediate and lasting improvement by correcting a minor bony incongruity which is the source of the patient’s presenting problem [22]. However, the capacity of MWM to reverse any positional fault remains unclear and further studies are required to assess the effectiveness of this technique.

The objective of this study is to determine the effect of MWM on anatomical and clinical characteristics of CAI, and to determine the long term effectiveness of this treatment.

The specific aims of the study are therefore:

1. To evaluate the effectiveness of MWM on clinically relevant outcomes, including patient-reported outcomes (dorsiflexion range, pain intensity, self- reported function, pressure pain threshold, static and dynamic balance), including long lasting benefits assessed at 12 months post treatment.
2. To assess the effect of MWM on changing the fibular position relative to the position of the tibia in CAI.

## Methods

### Design

This randomised controlled study has been registered in the Australian New Zealand Clinical Trial Registry (ANZTR) and ethical approval has been granted by the Human Research Ethics Committee of The University of Newcastle, Australia (H-2017-0354). Informed consent will be obtained in writing from all participants.

### Participants

Participants aged 18 years or over will be recruited from the general community in the Newcastle area of New South Wales, Australia through flyers posted on noticeboards in shopping centres, the University of Newcastle main campus, and various other public places. Recruitment advertising will also be via University of Newcastle social media channels. Volunteers with CAI will be accepted into the study if they satisfy the inclusion and exclusion criteria as endorsed by the International Ankle Consortium [29], except the time period for experiencing at least two episodes of giving way is changed from 6 months to 12 months to account for the seasonal nature of some sports (Table 1).

**Table 1:** Inclusion and exclusion criteria

Inclusion criteria	Exclusion criteria
• A history of at least one significant ankle sprain; - Initial sprain must have occurred at least 12 months prior to study enrolment - Was associated with inflammatory symptoms - Created at least one interrupted day of desired physical activity - The most recent injury must have occurred more than 3 months prior to study enrolment	• A history of previous surgeries to the musculoskeletal structures (i.e., bones, joint structures, nerves) in either lower extremity • A history of a fracture in either lower extremity requiring realignment • Acute injury to musculoskeletal structures of other joints of the lower extremity in the previous 3 months that impacted joint integrity and function (i.e., sprains, fractures), resulting in at least one interrupted day of desired physical activity • Have conditions for which manual therapy is generally contraindicated (such as the presence of a tumour, fracture, rheumatoid arthritis, osteoporosis, prolonged history of steroid use, or severe vascular disease)
• A history of the previously injured ankle joint ‘giving way’ and/or recurrent sprain and/or ‘feelings of instability’ - Participants should report at least two episodes of giving way in the 12 months prior to study enrolment	• Have conditions for which radiological imaging is contraindicated (e.g., pregnancy)
• Self-reported ankle instability should be confirmed with the Cumberland Ankle Instability Tool (CAIT) (≤24)	• Have conditions for which taping is contraindicated (e.g., allergy to strapping tape)
• General self-reported foot and ankle function questionnaire minimum score (Foot and Ankle Ability Measure (FAAM): activities of daily living (ADL) subscale < 90%, sport subscale < 80%)	• Receiving concurrent treatment - The most recent treatment for the ankle condition should have been received at least a week prior to study enrolment
	• Inability to read English

Data collection will be carried out at the physiotherapy and radiography research laboratories of the School of Health Sciences, The University of Newcastle, Australia.

### Procedure

This trial will adopt a pragmatic randomized controlled trial design to allow for real world application of MWM in a randomized setting [30]. This design has been used by previously published trials of manual therapy to better reflect routine clinical practice [31–33]. It enhances the external validity, but still controls for threats to internal validity.

The initial screening will be performed over the telephone after the potential participant contacts the research team. The screening questions are to determine if the potential participant meets some of the inclusion/exclusion criteria (Table 1). If a potential participant appears eligible following the telephone interview, further screening will be carried out using two standardised questionnaires: the Foot and Ankle Ability Measure (FAAM) [34], which measures function, and the Cumberland Ankle Instability Tool (CAIT) [35], which measures ankle instability. A link to access these questionnaires on the Qualtrics online survey platform (Qualtrics, Provo, Utah, USA) will be sent to the potential participant, along with the participant information statement and the consent form, through an email. Once the potential participant returns their completed forms, their final eligibility will be determined according to their scores (FAAM: activities of daily living (ADL) subscale < 90%, sport subscale < 80%; CAIT ≤24) on the two screening questionnaires. The participant will then be contacted to schedule an appointment for data collection.

Consenting participants will be randomised into two groups: an experimental group who will receive MWM, and a control group who will receive a placebo (detuned laser). All of the participants will be assessed for general joint hypermobility using the Beighton score [36]. Mechanical ankle instability will be tested separately for each ankle using an X-ray while undergoing an anterior drawer stress test [37, 38]. The clinically important outcome measures will include; radiological imaging of fibular position with respect to the tibia (positional fault), dorsiflexion range of motion (DFROM), pressure pain threshold (PPT), pain intensity, function, static balance and dynamic balance. These procedures and outcome measures are further explained below. The researcher who collects the clinical measurements, and the radiographer taking the X-rays, will be blinded to the participant’s group (intervention) allocation. This researcher will remain blinded to the group allocation until the 3 month follow-up. The 12 month follow-up data will be collected using online questionnaires.

Each participant will be randomly allocated to a group to receive either MWM (active) treatment or detuned laser treatment (placebo) (these interventions are fully explained below). The participant will be blinded as to whether they are receiving an active or placebo intervention, however due to the nature of the interventions, the treating practitioner cannot be blinded. Participants will be randomly allocated to groups according to a computer generated (GraphPad Software, Inc., CA, USA) randomisation schedule by another researcher not involved in data collection using sealed opaque envelopes. Each envelope will contain a piece of paper printed with either ‘1’ or ‘0’, for which ‘1’ denotes ‘MWM’ group and ‘0’ denotes ‘placebo’ group. The treating practitioner will open the envelope and allocate the participant to a group according to the number in the envelope, and deliver the designated treatment accordingly.

**Fig. 1:**
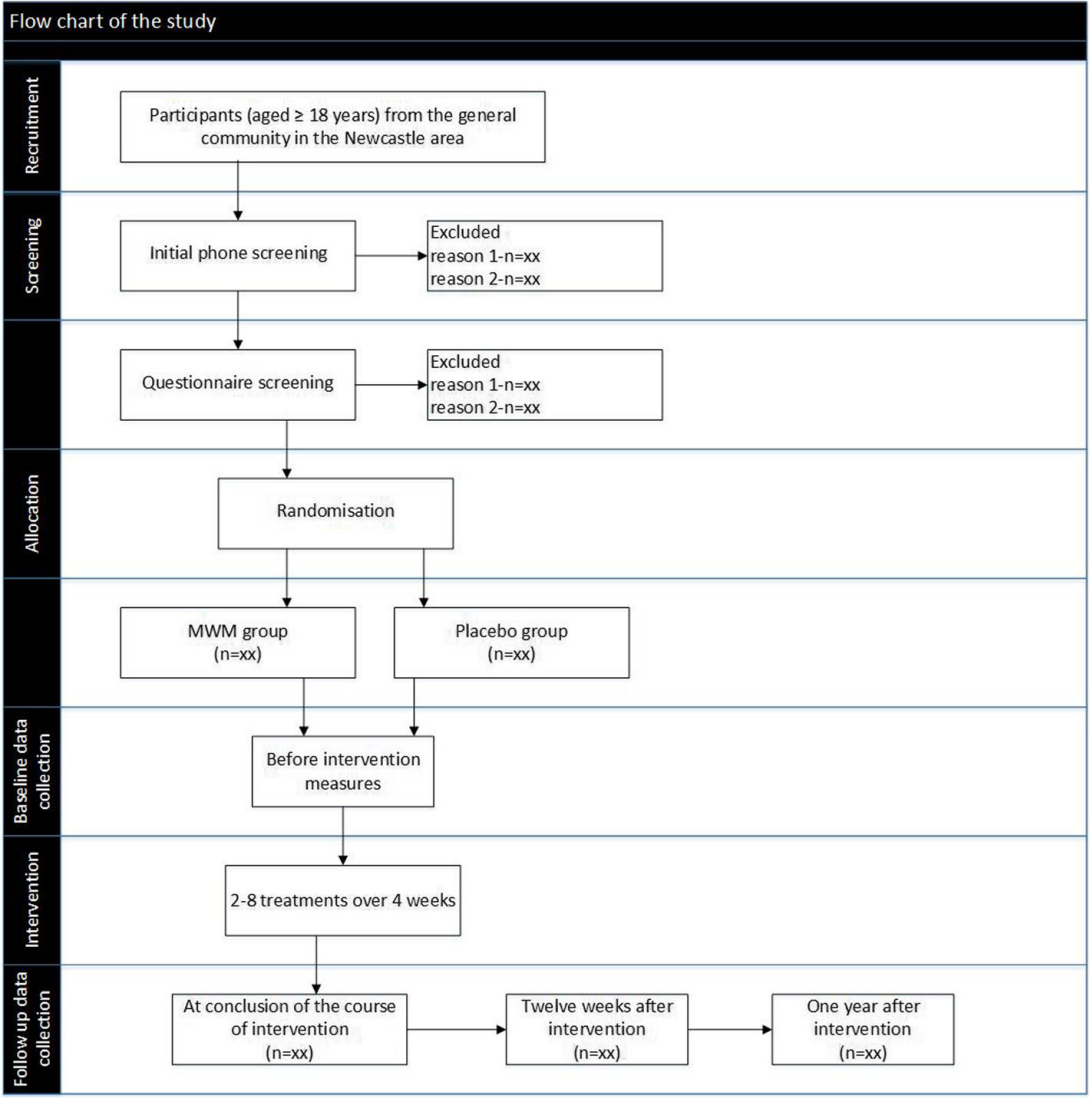
Flow of the study

Participants of both groups will attend for 2–8 treatment sessions over 4 weeks. The exact number of treatments needed to achieve an optimal change is not presently known, so a range allows the practitioner to exercise their clinical judgement. We have chosen two as the minimum number of treatments, because usual clinical practice would involve a minimum of two visits to enable re-assessment following the initial treatment [39]. The actual number of treatment sessions delivered to participants in each group will be determined according to the clinical judgement of the treating practitioner, who is a registered physiotherapist with a post-professional tertiary qualification in the field of manual therapy and more than 20 years of clinical experience in treating musculoskeletal conditions. The physiotherapist will also be individually instructed in the MWM intervention by an accredited member of the Mulligan Concept Teachers Association. The physiotherapist will conclude the course of intervention if the patient reports they have fully recovered or if no further improvement is possible up to a limit of eight sessions over a 4 week period. The number of sessions and the duration of each session will be recorded. The same measures taken at baseline will be repeated at the conclusion of the course of intervention, within a maximum of 4 days after the participant’s last intervention session. Further measurements will be repeated at the twelfth week with the exception of the imaging, and only self-report outcomes will be assessed at 12 months. Participants will be contacted by telephone every 4 weeks after finishing treatment for up to 1 year to record any new ankle injuries, any treatments undertaken, and their level of engagement in sport and other activities. Figure 1 describes the flow of the study.

### Outcome measures

#### Measurement of fibular positional fault from radiograph

A weight-bearing (neutral ankle in standing position) X-ray (55 k Vp and 2.1 mAs) will be taken of the affected ankle of the participant. The participant will be asked to stand on the foot to be imaged on a wooden box with the knee slightly flexed to simulate mid-stance of the gait cycle, with the foot of the non-stance leg hanging in a relaxed manner. The imaged leg will be maintained ~ 2 cm away and parallel to the image receptor. The same instructions will be given to all participants and the participant’s leg position will be monitored throughout the procedure. If any leg rotation is noted on imaging, the X-ray will be redone. The central ray will be centred at the base of the metatarsals and perpendicular to the image receptor, and the focal-film distance will be set to 110 cm. The participant will be allowed to hold on to body of the X-ray machine for balance if required.

**Fig. 2:**
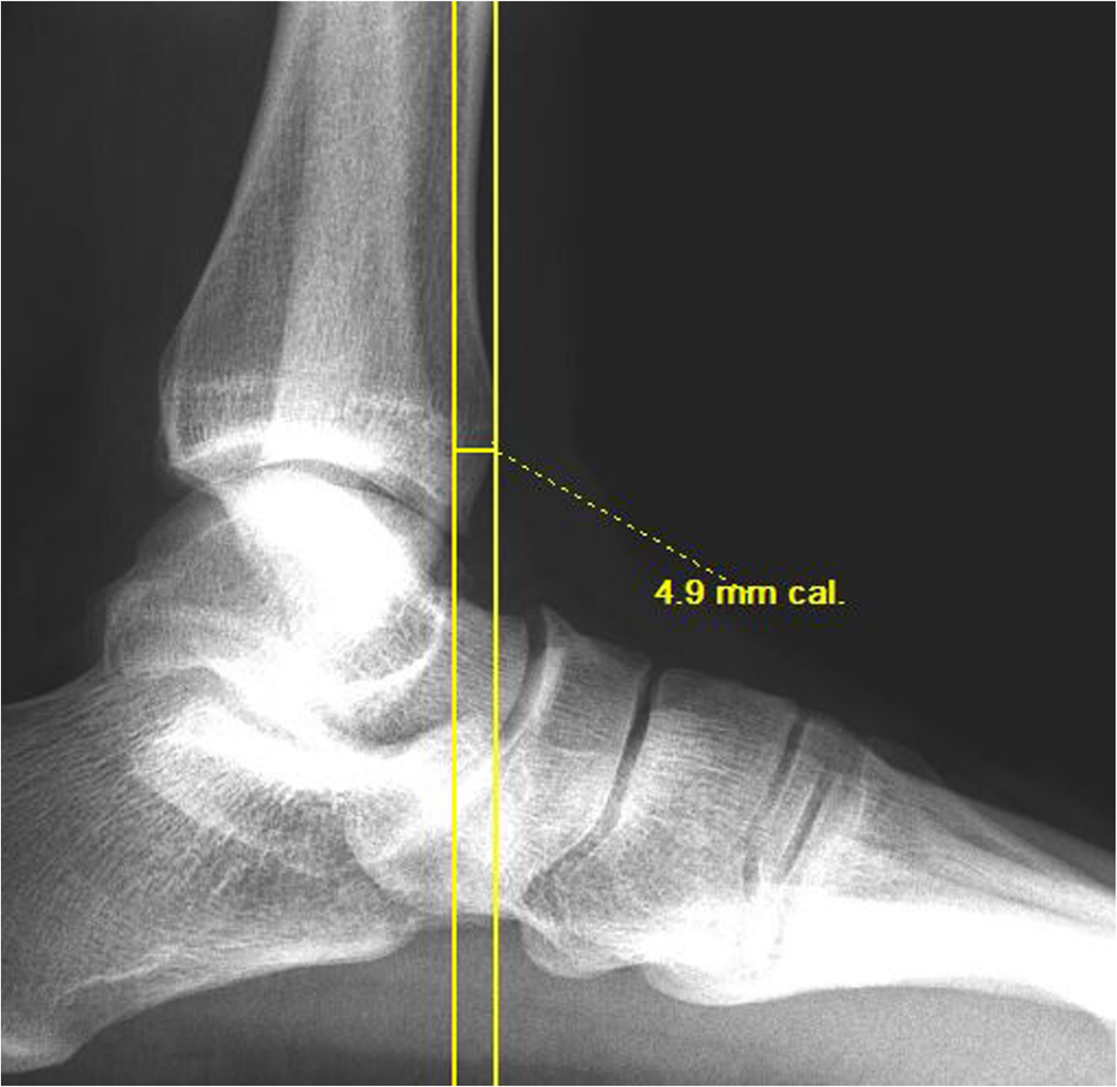
Fibular position measurement; the distance between the anterior edge of the distal fibula and the anterior edge of the distal tibia (right ankle, 4.2 mm in this image)

Radiographic images will be digitally obtained using Merge PACS™ software (Merge Health Care, 2012). The fibular position will be measured as the distance between the anterior edge of the distal fibula and the anterior edge of the distal tibia [27] (Fig. 2). The test-retest reliability intra-class correlation coefficient (ICC)_3,1_ has been estimated as 0.98, with a SEM of 0.64 mm for this measurement, and for intra-tester reliability, the ICC_3,1_ is 0.92 and SEM is 0.72 mm [27].

#### Weight-bearing dorsiflexion range of movement

Weight-bearing DFROM will be measured using the weight-bearing lunge test. The participant will be instructed to lunge towards the wall, touch their knee to the wall, and keep their heel in contact with the floor. Then the participant will be asked to move their foot away from the wall in 1 cm increments until the heel no longer maintains contact with the floor or the knee is no longer in contact with the wall. Maximal dorsiflexion will be considered to be the greatest distance between the great toe and wall with the participant’s knee maintaining contact with the wall [18, 40]. Both inter-rater reliability (ICC = 0.80–0.99) and intra-rater reliability (ICC = 0.65–0.99) have been reported as high for this test [41]. The same procedure will be followed for the opposite leg. Each centimetre away from the wall in the lunge test represents approximately 3.6 degrees of dorsiflexion [42]. Three test attempts will be performed and the average value will be used for analysis.

#### Pressure pain threshold

PPT measurements will be obtained in each leg from two local points (to assess local hypersensitivity) and one remote body area (to assess central sensitisation), in accordance with the method used in a previous study on acute ankle sprain [24]. The points include anterior to the lateral malleolus over the anterior talo-fibular ligament, inferior to the medial malleolus over the deltoid ligament, and over the proximal third of the tibialis anterior muscle belly.

A Freedom Tracker hand-held algometer (JTECH Medical, Salt Lake City, UT, USA) will be used for measuring PPT. A probe (contact surface of 1 cm^2^) will be placed perpendicular to the skin and pressure will be applied (40 kPa/s). The participant will be asked to indicate when the feeling of the stimulus changes from ‘pressure only’ to ‘discomfort’ by pressing an indicator switch [43, 44]. This process will be performed three consecutive times and a 10 s rest period will be allowed between each set of measurements. Pressure algometry is considered a stable and reliable measure of PPT [45]. The inter-rater reliability of pressure algometry has been reported to be high when the algometer pressure is applied at a consistent rate (ICC 0.91, 95% CI 0.82–0.97) [46].

#### Pain intensity

Current pain intensity will be assessed using the visual analogue scale (VAS) which consists of a 100 mm horizontal line, with ‘no pain’ anchored on the left of the line and ‘worst possible pain’ anchored on the right. The validity of the VAS for detecting changes in pain intensity has been supported by several studies [47, 48].

The participant will also be asked to indicate all areas in which they currently feel symptoms on a body chart. The areas in which they feel ‘pain’ will be shaded; the areas in which they feel ‘tingling, pricking, or burning’ will be circled; and the areas where they feel ‘numbness, heaviness or other sensations’ will be indicated on the chart by an ‘N’.

#### Function

Self-reported physical function of the participant will be evaluated using the FAAM which consists of a 21-item ADL subscale and an 8-item sport subscale [34]. This tool has been documented as a reliable, responsive and valid measure of physical function for individuals with a broad range of musculoskeletal disorders of the lower leg, foot and ankle [34]. The Foot and Ankle Outcome Score (FAOS) questionnaire comprising 42 items will also be used, and has been reported as also being a reliable and valid measure (ICCs reported as 0.78, 0.86, 0.70, 0.85, 0.92 for the five subscales of pain, symptoms, ADL, sport and recreation function, and foot- and ankle-related quality of life, respectively) [49].

Further, the participant will be asked to identify up to three important activities that they are unable to perform or are having moderate to extreme difficulty performing due to pain. For each activity, the participant will be asked to rate between 0 and 10 the level of difficulty they experience performing that activity using the Patient-Specific Functional Scale (PSFS) [35]. The construct validity of the PSFS is well supported, and the test-retest reliability has been assessed as moderate to good (ICC2,1 = 0.713) [36].

#### Static balance

For static balance, the participant will stand barefoot on the centre of a force plate (KISTLER 9260AA6, Winterthur, Switzerland), assuming a standardized single leg stance position. The participant will then be instructed to flex the other leg slightly at the hip, with the knee flexed to 90 degrees. Their arms will be crossed at their chest with each hand resting on the opposite shoulder. Measurements will be recorded with both ‘eyes open’ and ‘eyes closed’. For ‘eyes open’, the participant will be asked to maintain a fixed gaze on a cross marked on the wall three metres in front of them and remain as still as possible for 10 s [50]. For ‘eyes closed’, the participant will be asked to close their eyes and remain as still as possible for 10 s [50]. If the participant is unable to stand for 10 s, the standing time achieved will be recorded. Only averaged Centre of Pressure (CoP) data including sway velocity, sway area per second, sway average amplitude and sway maximal amplitude will be used in the analysis to maintain consistency. CoP data obtained through the force platform will be acquired at 100 Hz [21].

#### Dynamic balance

Dynamic balance will be assessed using the Star Excursion Balance Test (SEBT) which has been shown to be a reliable measure to identify dynamic balance deficits in patients with a variety of lower extremity conditions [51]. The participant will be asked to establish a stable base of support on the stance limb in the middle of the testing grid on a force plate (KISTLER 9260AA6, Winterthur, Switzerland). While standing on a single limb, the participant will be asked to reach as far as possible with the reaching limb along each line (anterior, postero-medial and postero-lateral directions), lightly touching the line with the most distal portion of the reaching foot without shifting weight or coming to rest on the foot of the reaching limb. The participant will then be asked to return the reaching limb to the starting position in the centre of the grid. If the individual lifts or shifts any part of the foot of the stance limb during the trial, the trial will be not considered as complete [51].

After performing a maximum of four non-recorded trials for familiarisation, the next trial for each direction will be recorded for the purpose of analysis [52, 53]. Normalised SEBT values will be obtained by dividing the excursion distance by the participant’s leg length (the distance between the anterior superior iliac spine and the ipsilateral medial malleolus), and then multiplying by 100 [52, 54]. Data for centre of pressure (CoP) velocity (V) to quantify spatio-temporal parameters (VCoP-total, VCoP-mediolateral, VCoP-anteroposterior) will be acquired at 100 Hz, under the foot during unipodal stance [52].

### Perceptions of the credibility of the placebo

At the data collection session at the conclusion of course of intervention, the participant will be asked to indicate which intervention (active or placebo) they thought they had received during the last 4 weeks and to give a confidence rating on a scale of 0–10 (with 0=‘not at all confident’ and 10 = ‘extremely confident’ [55]). Global perceived effect will also be measured using a self-assessment of improvement on a seven point rating scale (1 = completely recovered, 2 = much improved, 3 = slightly improved, 4 = not changed, 5 = slightly worsened, 6 = much worsened, 7 = worse than ever) in response to the question ‘How would you rate the course of your ankle complaints since the start of this study?’ [56, 57].

### Other measures

Telephone interviews will be conducted monthly after enrolment up to 1 year to record any new injuries, any treatments undertaken, and the level of engagement in sports and other activities. These variables will be used as covariates in the analysis of the 12 month follow-up data as they are possible confounders. Further, the Beighton score for hypermobility and radiographic measurement of the anterior drawer stress test will be recorded.

#### Beighton score

Scoring for joint hypermobility will be undertaken according to previously published methods [36]. Each participant will be assessed in five test positions, as follows:

1. Passive extension of the fifth metacarpophalangeal (MCP) joint to ≥90 degrees. The participant sits on a chair at the short side of the table with the shoulder in 80 degrees abduction, elbow flexed at 90 degrees, and the forearm resting on the table in a pronated position. The fifth MCP joint is passively extended by the researcher and a goniometer is used to measure the angle.
2. Passive hyperextension of the elbow ≥10 degrees. The participant sits on a chair with the shoulder at 90 degrees of flexion and the forearm supinated. A goniometer is placed at the lateral epicondyle and the measurement is taken at maximum elbow extension.
3. Passive hyperextension of the knee ≥10 degrees. The participant lies supine with their legs in the horizontal plane. The goniometer is placed at the lateral femoral condyle and the measurement taken at maximum knee extension.
4. Passive apposition of the thumb to the flexor side of the forearm. The score is positive if the entire thumb touches the flexor side of the forearm while the shoulder is flexed at 90 degrees, the elbow extended, and the forearm pronated.
5. Forward flexion of the trunk with the knees straight. The score is positive if the participant’s hand palms rest easily on the floor.

#### Anterior drawer stress test with radiographic measurement

Ankle joint mechanical instability will be assessed using a lateral x-ray to measure the amount of anterior translation of the talus during a ligament stress test for each ankle. The radiograph will be taken while the ankle is undergoing a simulated anterior drawer test using 125 N force [38]. The stress radiograph will be taken with the participant in a supine lying position with the foot relaxed in a resting position and the lower leg resting on a support, with the hip and knee each flexed approximately 45 degrees. The heel will be supported on a dynamometer (Lafayette Manual Muscle Tester, Model 01165, Lafayette, IN, USA) attached to a customised device which produces the anteriorly directed force. The distal tibia will be fixed on the support using a stabilising belt placed over the distal aspect of the tibia [58]. The central ray will be centred just above the tip of the lateral malleolus and perpendicular to the image receptor [59]. Then an anterior force of 125 N will be applied [38] to the heel of the participant at an angle of 20 degrees to the vertical plane as per recommended clinical practice [60], using the customised device. The force will be monitored using the digital display of the dynamometer attached to the customised device, and the radiograph will be taken at 125 N. The ankle radiograph will be taken at the focal-film distance of 110 cm [61] and will set to 55 kVp and 2.1 mAs. The same procedure will be applied to the other ankle. These images will be taken at the baseline data collection session to assess mechanical instability for use in subgroup analysis.

**Fig. 3:**
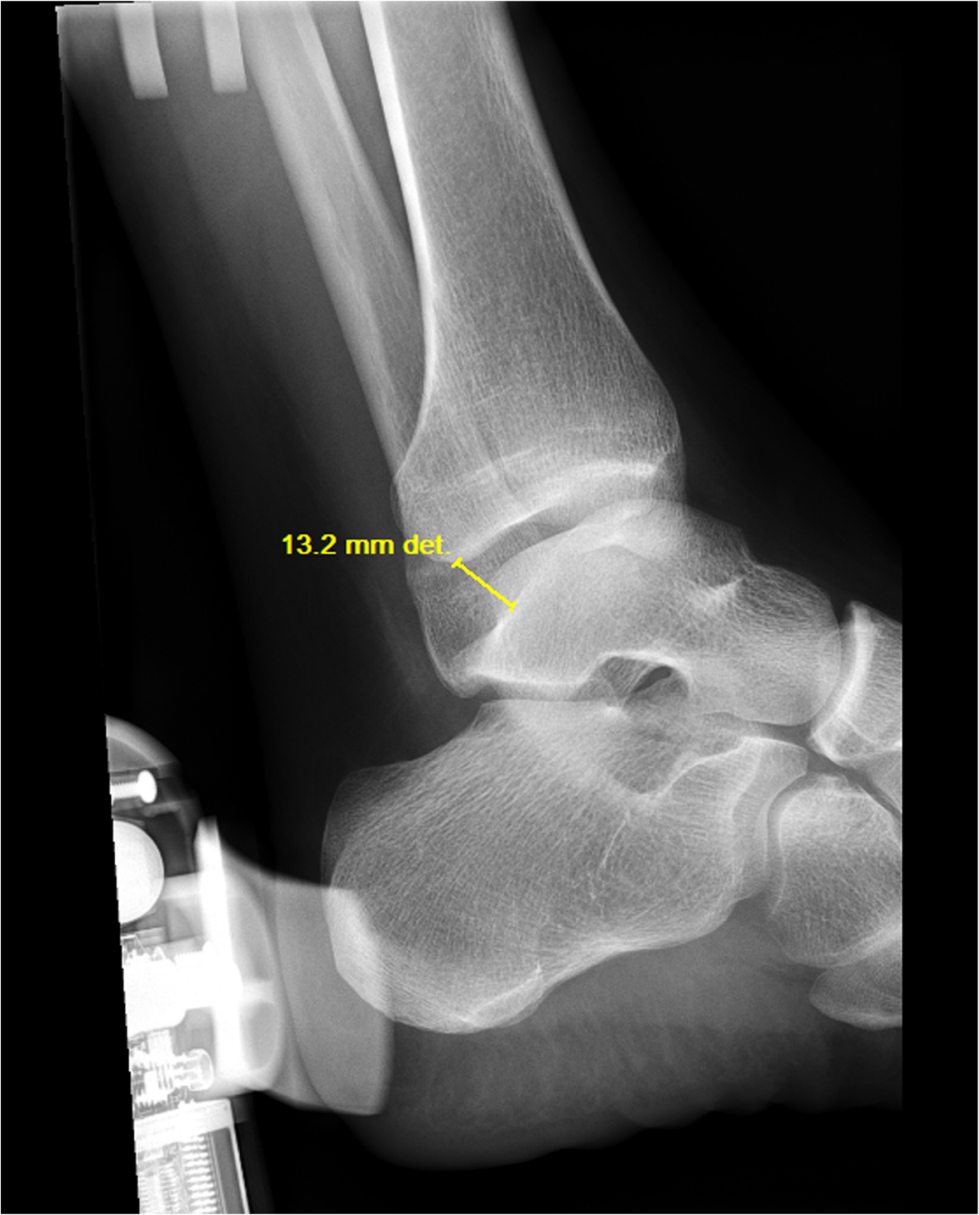
Anterior translation of the talus during the anterior drawer stress test is measured as the distance on X-ray from the posterior lip of the tibial joint surface to the nearest point of the talar dome (left ankle, 13.2 mm in this image)

Radiographic images will be digitally obtained using Merge PACS™ software (Merge Health Care, 2012). Anterior translation of the talus will be measured between the posterior lip of the tibial articular surface and the nearest point of the talar dome (Fig. 3) [61–64] to identify any mechanical instability. Anterior drawer stress radiographs have been found to have moderate sensitivity, high specificity and a high positive predictive value for the evaluation of lateral ankle instability [37]. A between-limb difference of 3 mm in anterior translation of the talus or an absolute value of 10 mm is considered clinically significant [37].

### Application of the intervention

Participants in the experimental group will be treated with a manual MWM technique to the ankle and will be taped after the intervention using the Mulligan approach [22] to attempt to maintain the effects of the MWM. The control group will receive a detuned (inactive) therapeutic laser treatment to the lateral region of the ankle. The number of treatment sessions delivered for each participant will be based on their symptomatic response to treatment, as determined by the clinical judgement of the treating practitioner. Each participant will be asked to avoid concurrent interventions during their participation in the study.

### MWM intervention

The participant’s inferior tibio-fibular joint will be mobilised using Mulligan’s fibula MWM for dorsiflexion and/or inversion [22]. Initially, the technique will be performed in supine lying with the tibia resting on the treatment table and the foot unsupported off the table’s edge. The practitioner applies a sustained pain-free anteroposterior glide with a slight cephalad and lateral inclination to the distal fibula (lateral malleolus). This glide is maintained while the participant performs active inversion or dorsiflexion (depending on which is more limited in range) to end of range. There should be no pain with the active movement. At the end of range, the practitioner will apply and sustain overpressure to the active movement for a few seconds (or the participant will do so after appropriate instruction) [16, 22]. If dorsiflexion remains restricted, this technique can be progressed and performed in partial and/or full weight-bearing.

One treatment session will consist of three to five sets, with six to ten repetitions of the active movement in each set, with the actual dosage depending on the individual response of the participant [22]. Participants will receive between two to eight sessions according to the clinical reasoning of the practitioner, over a period of 4 weeks. After each session, Mulligan MWM taping will be applied in an attempt to replicate the sustained fibula glide [8]. Non-elastic tape will be applied to the ankle starting 2 cm anterior to the fibula and 1 cm proximal to the tip of the lateral malleolus. The tape will be spiralled obliquely around the lower limb while the fibula glide is sustained, finishing on the anterior aspect of the leg [22]. The participant will be instructed to keep the tape on for 24 h. In the case of an adverse reaction, they will be advised to remove the tape immediately and note the length of time the tape was in place.

### Detuned laser intervention

The placebo intervention will be applied using a detuned therapeutic laser device (Meyer Medical Electronics, Mordialloc, Australia) for 5 min to the lateral region of the ankle, maintaining the probe 0.5–1 cm away from the skin [31, 65–67]. The detuned laser device will appear to function normally (both audibly and visually) to participants, but no effective emission will be produced. Both the participant and the practitioner will be required to wear protective glasses as per normal clinical practice [66, 68]. Participants will receive two to eight treatments over 4 weeks, according to the clinical judgement of the treating practitioner. Detuned laser has been used in several other studies assessing manual therapy including for chronic ankle sprains. It avoids any possible direct mechanical effects to the ankle being treated and also does not activate somatosensory receptors [69–71]. Further, it has been shown to have a strong placebo effect [70]. Scheduling of participant appointments will be arranged to avoid interaction between participants.

## Sample size and data analysis

Previously published data related to the primary outcome measure of function (FAAM subscales, ADL and sports) [18, 34] (MCID = 8.0, SD = 5.68; MCID = 9.0, SD = 7.42 respectively) were used in sample size calculations [18, 34, 72]. A sample size of 16 per group allowing for a 30% drop-out rate was estimated, for a minimal statistical power of 0.80 and an alpha significance level of 0.05. Secondary analysis based on the subgroups of ankle instability (mechanical, functional) will be preliminary in nature as the study is not powered for this aim.

Data will be analysed using SPSS Statistics for Windows (Version 23.0, Armonk, NY, IBM Corp). Continuous data will be assessed for normality using the Shapiro-Wilk test.

Baseline comparability between groups will be analysed using the independent t-test or non-parametric equivalent, as appropriate. Linear mixed models will be used to analyse the outcome measures. For the primary outcome measure, ‘function’ will be the outcome variable and time, group and an interaction term for time by group will be the predictors. Any statistically significant difference in change in the outcome variable over time between the groups will be indicated by the *p* value for the interaction term. Pairwise Bonferroni comparisons will be performed to explore the differences between time points and between groups if a significant interaction is identified. Independent t-tests will be used to compare outcome measures between groups at each time point and the changes of the scores will be used to detect any changes in the outcomes of interest. Intention to treat analysis will be performed with all participants allocated to each group condition to evaluate the effect of the independent variable. For missing data in ITT analysis, a participant’s last observation for each outcome measure will be carried forward. The average number and the average duration of intervention sessions between groups will be compared. If any significant difference observed, secondary analysis will be taken to find any correlation between the treatment volume and the outcomes.

Additional variables recorded during monthly phone interviews (new injuries, changes in activity level, and occurrence of other treatments) will be used as covariates in the analysis of the 12 month follow-up data as they are possible confounders. Further, the Beighton score for hypermobility will also be included in regression analysis as a covariate.

Radiographic measurement of the anterior drawer stress test will be used to differentiate subgroups of CAI in potential sub group analysis.

## Discussion

One proposed anatomical mechanism underpinning MWM is theorised to be a correction of a minor bony incongruity (positional fault) which is at the source of the patient’s presenting problem [22, 73]. The existence of an anterior fibular ‘positional fault’ in individuals with CAI has some preliminary radiological support [27]. There are also limited MRI data supporting Mulligan’s positional fault hypothesis in cases of lateral ankle pain [72], however there is no evidence to date that MWM reverses any positional anomaly. Further, should any fibular positional anomaly be reversed immediately after the application of MWM, the length of time this reversal or correction is maintained is unknown. The proposed study protocol is designed to determine the presence of any positional fault of the fibula in CAI, and whether MWM can reverse this, and if so, whether this reversal is evident 4 weeks after treatment commences. Moreover, this study protocol will explore the correlation between an anatomical measure (fibular position) and other clinical outcomes (pain, function, pressure pain threshold, DFROM, static and dynamic balance). Potential relationships between these measures may help explain how changing an anatomical measure may effect a clinically meaningful outcome. The effect of MWM in CAI will also be explored in relation to the presence or not of radiologically measurable mechanical instability.

There are very few clinical trials with long term follow-ups which have assessed MWM for any musculoskeletal condition, and only one for CAI which had a 6 month follow-up [6, 15]. The proposed study protocol is therefore the first designed to evaluate the long term effectiveness of MWM on CAI. Moreover, the treatment effect may depend on the type of instability present (mechanical or functional), and this study protocol may evaluate the efficacy of MWM on these two subgroups of CAI. However, the subgroup analysis will be exploratory as the study was only powered to detect the main effect being the intervention on the functional outcome.

## Abbreviations

ADL: Activities of daily living
ANZCTR: Australian New Zealand Clinical Trial Registry
AP: Antero-posterior
CAI: Chronic ankle instability
CAIT: Cumberland Ankle Instability Tool
CoP: Centre of pressure
DFROM: Dorsiflexion range of motion
FAAM: Foot and Ankle Ability Measure
FAOS: Foot and Ankle Outcome Score
MCID: Minimal clinically important difference
MCP: Metacarpophalangeal
ML: Medio-lateral
MWM: Mobilisation With Movement
PPT: Pressure pain threshold
PSFS: Patient-Specific Functional Scale
SD: Standard deviation
SEBT: Star Excursion Balance Test
VAS: Visual Analogue Scale

## References

[1] Hershkovich O, Tenenbaum S, Gordon B, Bruck N, Thein R, Derazne E, Tzur D, Shamiss A, Afek A (2015). A large-scale study on epidemiology and risk factors for chronic ankle instability in young adults. J Foot Ankle Surg.

[2] Doherty C, Delahunt E, Caulfield B, Hertel J, Ryan J, Bleakley C (2014). The incidence and prevalence of ankle sprain injury: a systematic review and meta-analysis of prospective epidemiological studies. Sports Med.

[3] Gigi R, Haim A, Luger E, Segal G, Melamed E, Beer Y, Nof M, Nyska M, Elbaz A (2015). Deviations in gait metrics in patients with chronic ankle instability: a case control study. J Foot Ankle Res.

[4] Hertel J (2002). Functional anatomy, Pathomechanics, and pathophysiology of lateral ankle instability. J Athl Train.

[5] Martin RL, Davenport TE, Paulseth S, Wukich DK, Godges JJ (2013). Ankle stability and movement coordination impairments: ankle ligament sprains. J Orthop Sports Phys Ther.

[6] Weerasekara I, Osmotherly P, Snodgrass S, Marquez J, de Zoete R, Rivett DA (2018). Clinical Benefits of Joint Mobilization on Ankle Sprains: A Systematic Review and Meta-Analysis. Arch Phys Med Rehabil.

[7] Pettman E (2007). A history of manipulative therapy. J Man Manip Ther.

[8] Vicenzino B, Hing W, Rivett DA, Hall T. Mobilisation with movement: the art and the science. Australia: Elsevier; 2011.

[9] Mau H, Baker RT (2014). A modified mobilization-with-movement to treat a lateral ankle sprain. Int J Sports Phys Ther.

[10] McCarthy C, Bialosky J, Rivett D. Spinal manipulation. In: Jull G, Moore A, Falla D, Lewis J, McCarthy C, Sterling M, editors. Grieve's Modern Musculoskeletal Physiotherapy. 4th edn: Australia: Elsevier; 2015. p. 278–82.

[11] Chien A, Eliav E, Sterling M (2009). Hypoaesthesia occurs with sensory hypersensitivity in chronic whiplash--further evidence of a neuropathic condition. Man Ther.

[12] Sterling M, Jull G, Wright A (2001). Cervical mobilisation: concurrent effects on pain, sympathetic nervous system activity and motor activity. Man Ther.

[13] Beazell JR, Grindstaff TL, Sauer LD, Magrum EM, Ingersoll CD, Hertel J (2012). Effects of a proximal or distal tibiofibular joint manipulation on ankle range of motion and functional outcomes in individuals with chronic ankle instability. J Orthop Sports Phys Ther.

[14] Reid A, Birmingham TB, Alcock G (2007). Efficacy of mobilization with movement for patients with limited dorsiflexion after ankle sprain: a crossover trial. Physiotherapy Canada.

[15] Cruz-Diaz D, Lomas Vega R, Osuna-Perez MC, Hita-Contreras F, Martinez-Amat A (2015). Effects of joint mobilization on chronic ankle instability: a randomized controlled trial. Disabil Rehabil.

[16] Vicenzino B, Branjerdporn M, Teys P, Jordan K (2006). Initial changes in posterior talar glide and dorsiflexion of the ankle after mobilization with movement in individuals with recurrent ankle sprain. J Orthop Sports Phys Ther.

[17] Marron-Gomez D, Rodriguez-Fernandez A, Martin-Urrialde J (2015). The effect of two mobilization techniques on dorsiflexion in people with chronic ankle instability. Phys Ther Sport.

[18] Gilbreath JP, Gaven SL, Van Lunen L, Hoch MC (2014). The effects of mobilization with movement on dorsiflexion range of motion, dynamic balance, and self-reported function in individuals with chronic ankle instability. Man Ther.

[19] Someeh M, Norasteh AA, Daneshmandi H, Asadi A (2015). Influence of Mulligan ankle taping on functional performance tests in healthy athletes and athletes with chronic ankle instability. Int J Athl Ther Train.

[20] Someeh M, Norasteh AA, Daneshmandi H, Asadi A (2015). Immediate effects of Mulligan's fibular repositioning taping on postural control in athletes with and without chronic ankle instability. Phys Ther Sport.

[21] Hopper D, Samsson K, Hulenik T, Ng C, Hall T, Robinson K (2009). The influence of Mulligan ankle taping during balance performance in subjects with unilateral chronic ankle instability. Phys Ther Sport.

[22] Hing W, Hall T, Rivett DA, Vicenzino B, Mulligan B (2015). The Mulligan concept of manual therapy.

[23] Adal SA, Pourkazemi F, Mackey M, Hiller C (2017). Presence of pain in people with chronic ankle instability. Br J Sports Med.

[24] Ramiro-Gonzalez MD, Cano-de-la-Cuerda R, De-la-Llave-Rincon AI, Miangolarra-Page JC, Zarzoso-Sanchez R, Fernandez-de-Las-Penas C (2012). Deep tissue hypersensitivity to pressure pain in individuals with unilateral acute inversion ankle sprain. Pain Med.

[25] Collins N, Teys P, Vicenzino B (2004). The initial effects of a Mulligan's mobilization with movement technique on dorsiflexion and pain in subacute ankle sprains. Man Ther.

[26] Mulligan BR (1995). Manual therapy: NAGS, SNAGS, MWMs, etc 6th ed edn.

[27] Hubbard TJ, Hertel J, Sherbondy P (2006). Fibular position in individuals with self-reported chronic ankle instability. J Orthop Sports Phys Ther.

[28] Hoch MC, Mullineaux DR, Andreatta RD, English RA, Medina-McKeon JM, Mattacola CG, McKeon PO (2014). Effect of a 2-week joint mobilization intervention on single-limb balance and ankle arthrokinematics in those with chronic ankle instability. J Sport Rehabil.

[29] Gribble PA, Delahunt E, Bleakley C, Caulfield B, Docherty C, Fourchet F, Fong D, Hertel J, Hiller C, Kaminski T (2013). Selection criteria for patients with chronic ankle instability in controlled research: a position statement of the international ankle consortium. J Orthop Sports Phys Ther.

[30] Alsop J, Scott M, Archey W (2016). The mixed randomized trial: combining randomized, pragmatic and observational clinical trial designs. J Comp Effect Res.

[31] Reid SA, Callister R, Snodgrass SJ, Katekar MG, Rivett DA (2015). Manual therapy for cervicogenic dizziness: long-term outcomes of a randomised trial. Man Ther.

[32] Groeneweg R, van Assen L, Kropman H, Leopold H, Mulder J, Smits-Engelsman BCM, Ostelo RWJG, Oostendorp RAB, van Tulder MW (2017). Manual therapy compared with physical therapy in patients with non-specific neck pain: a randomized controlled trial. Chiropr Man Therap.

[33] Deyle GD, Gill NW, Rhon DI, et al. A multicentre randomised, 1-year comparative effectiveness, parallel-group trial protocol of a physical therapy approach compared to corticosteroid injections. BMJ Open. 2016;6:e010528. 10.1136/bmjopen-2015-010528 PMC4823390 27033961

[34] Martin RL, Irrgang JJ, Burdett RG, Conti SF, Van Swearingen JM (2005). Evidence of validity for the foot and ankle ability measure (FAAM). Foot Ankle Int.

[35] Hiller CE, Refshauge KM, Bundy AC, Herbert RD, Kilbreath SL (2006). The Cumberland ankle instability tool: a report of validity and reliability testing. Arch Phys Med Rehabil.

[36] Smits-Engelsman B, Klerks M, Kirby A (2011). Beighton score: a valid measure for generalized hypermobility in children. J Pediatr.

[37] Jolman S, Robbins J, Lewis L, Wilkes M, Ryan P. Comparison of magnetic resonance imaging and stress radiographs in the evaluation of chronic lateral ankle instability. Foot Ankle Int. 2017;1071100716685526. 10.1177/1071100716685526 28061547

[38] Hubbard TJ, Cordova M (2009). Mechanical instability after an acute lateral ankle sprain. Arch Phys Med Rehabil.

[39] Maitland GD (2005). Maitland's vertebral manipulation, 7 edn.

[40] Hoch MC, Andreatta RD, Mullineaux DR, English RA, Medina McKeon JM, Mattacola CG, McKeon PO (2012). Two-week joint mobilization intervention improves self-reported function, range of motion, and dynamic balance in those with chronic ankle instability. J Orthop Res.

[41] Powden CJ, Hoch JM, Hoch MC (2015). Reliability and minimal detectable change of the weight-bearing lunge test: a systematic review. Man Ther.

[42] Bennell KL, Talbot RC, Wajswelner H, Techovanich W, Kelly DH, Hall AJ (1998). Intra-rater and inter-rater reliability of a weight-bearing lunge measure of ankle dorsiflexion. Aust J Physiother.

[43] Arendt-Nielsen L, Nie H, Laursen MB, Laursen BS, Madeleine P, Simonsen OH, Graven-Nielsen T (2010). Sensitization in patients with painful knee osteoarthritis. PAIN.

[44] Rebbeck T, Moloney N, Azoory R, Hubscher M, Waller R, Gibbons R, Beales D (2015). Clinical ratings of pain sensitivity correlate with quantitative measures in people with chronic neck pain and healthy controls: cross-sectional study. Phys Ther.

[45] Frank L, McLaughlin P, Vaughan B (2013). The repeatability of pressure algometry in asymptomatic individuals over consecutive days. Int J Osteopath Med.

[46] Chesterton LS, Sim J, Wright CC, Foster NE (2007). Interrater reliability of algometry in measuring pressure pain thresholds in healthy humans, using multiple raters. Clin J Pain.

[47] Ferreira-Valente MA, Pais-Ribeiro JL, Jensen MP (2011). Validity of four pain intensity rating scales. Pain.

[48] Price DD, McGrath PA, Rafii A, Buckingham B (1983). The validation of visual analogue scales as ratio scale measures for chronic and experimental pain. Pain.

[49] Roos EM, Brandsson S, Karlsson J (2001). Validation of the foot and ankle outcome score for ankle ligament reconstruction. Foot Ankle Int.

[50] Trojian TH, McKeag DB (2006). Single leg balance test to identify risk of ankle sprains. Br J Sports Med.

[51] Gribble PA, Hertel J, Plisky P (2012). Using the star excursion balance test to assess dynamic postural-control deficits and outcomes in lower extremity injury: a literature and systematic review. J Athl Train.

[52] Pionnier R, Decoufour N, Barbier F, Popineau C, Simoneau-Buessinger E (2016). A new approach of the star excursion balance test to assess dynamic postural control in people complaining from chronic ankle instability. Gait Posture.

[53] Robinson RH, Gribble PA (2008). Support for a reduction in the number of trials needed for the star excursion balance test. Arch Phys Med Rehabil.

[54] Gribble PA, Hertel J (2003). Considerations for normalizing measures of the star excursion balance test. Meas Phys Educ Exerc Sci.

[55] Owens JE, Menard M (2011). The quantification of placebo effects within a general model of health care outcomes. J Altern Complement Med.

[56] van der Windt DA, Koes BW, Deville W, Boeke AJ, de Jong BA, Bouter LM (1998). Effectiveness of corticosteroid injections versus physiotherapy for treatment of painful stiff shoulder in primary care: randomised trial. Bmj.

[57] Kamper SJ, Ostelo RWJG, Knol DL, Maher CG, de Vet HCW, Hancock MJ (2010). Global Perceived Effect scales provided reliable assessments of health transition in people with musculoskeletal disorders, but ratings are strongly influenced by current status. J Clin Epidemiol.

[58] Seebauer CJ, Bail HJ, Rump JC, Hamm B, Walter T, Teichgräber UKM (2013). Ankle laxity: stress investigation under MRI control. Am J Roentgenol.

[59] Johannsen A (1978). Radiological diagnosis of lateral ligament lesion of the ankle. A comparison between talar tilt and anterior drawer sign. Acta Orthop Scand.

[60] Dutton M (2017). Dutton's Orthopaedic Examination, Evaluation, and Intervention, 4thg edn.

[61] Lee KM, Chung CY, Kwon S-S, Chung MK, Won SH, Lee SY, Park MS (2013). Relationship between stress ankle radiographs and injured ligaments on MRI. Skelet Radiol.

[62] Beynnon BD, Webb G, Huber BM, Pappas CN, Renström P, Haugh LD (2005). Radiographic measurement of anterior talar translation in the ankle: determination of the most reliable method. Clin Biomech.

[63] Prado MP, Fernandes TD, Camanho GL, Mendes AAM, Amodio DT (2013). Mechanical instability after acute ankle ligament injury: randomized prospective comparison of two forms of conservative treatment. Rev Bras Ortop.

[64] Ellis SJ, Williams BR, Pavlov H, Deland J (2011). Results of anatomic lateral ankle ligament reconstruction with tendon allograft. HSS J.

[65] Teo TKB, Tay KH, Lin SE, Tan SG, Lo RH, Taneja M, Irani FG, Sebastien MG, Lim KH, Tan BS. Endovenous Laser Therapy in the Treatment of Treatment of Lower-limb Venous Ulcers. J Vasc Interv Radiol. 2010;21(5):657–662. 10.1016/j.jvir.2010.01.029 20430295

[66] de Bie RA, de Vet HC, Lenssen TF, van den Wildenberg FA, Kootstra G, Knipschild PG. Low-level laser therapy in ankle sprains: a randomized clinical trial. Arch Phys Med Rehabil. 1998;79(11):1415–20. 10.1016/s0003-9993(98)90237-4 9821903

[67] Kingsley JD, Demchak T, Mathis R (2014). Low-level laser therapy as a treatment for chronic pain. Front Physiol.

[68] Cotler HB, Chow RT, Hamblin MR, Carroll J (2015). The use of low level laser therapy (LLLT) for musculoskeletal pain. MOJ Orthop Rheumatol.

[69] Irnich D, Behrens N, Molzen H, Konig A, Gleditsch J, Krauss M, Natalis M, Senn E, Beyer A, Schops P (2001). Randomised trial of acupuncture compared with conventional massage and "sham" laser acupuncture for treatment of chronic neck pain. Bmj.

[70] Reid SA, Rivett DA, Katekar MG, Callister R (2008). Sustained natural apophyseal glides (SNAGs) are an effective treatment for cervicogenic dizziness. Man Ther.

[71] Pellow JE, Brantingham JW (2001). The efficacy of adjusting the ankle in the treatment of subacute and chronic grade I and grade II ankle inversion sprains. J Manip Physiol Ther.

[72] Merlin DJ, McEwan IM, Thom JM (2005). Mulligan's mobilisation with movement technique for lateral ankle pain and the use of magnetic resonance imaging to evaluate the "positional fault" hypothesis.

[73] Vicenzino B, Paungmali A, Teys P (2007). Mulligan's mobilization-with-movement, positional faults and pain relief: current concepts from a critical review of literature. Man Ther.

